# Investigation of minocycline and hyaluronic acid combined with ultrasound therapy in a Staphylococcus aureus-infected rat wound model

**DOI:** 10.1099/mic.0.001612

**Published:** 2025-10-08

**Authors:** Yu Gou, Yi Zhang, Liangjia Bi, Jiapin Zou, Dian Yu, Deshu Zhuang

**Affiliations:** 1 Department of Stomatology, The Fourth Affiliated Hospital, Harbin Medical University, Harbin 150001, PR China; 2 Department of Stomatology, Beijing Chao-Yang Hospital, Capital Medical University, Beijing 100020, PR China; 3 Department of Stomatology, Sanya Central Hospital Affiliated to Hainan Medical College, Sanya 572022, PR China; 4 Heilongjiang Research Center of Molecular Medicine Engineering Technology (No. GCJSYJZX20200099), Harbin 150001, PR China

**Keywords:** hyaluronic acid, minocycline, *Staphylococcus aureus*, ultrasound, wound infection

## Abstract

This study aimed to examine the effects of minocycline (MINO) and hyaluronic acid (HA) on wound healing in rats. MINO/HA was combined with ultrasound therapy for treating wounds infected with *Staphylococcus aureus*. Cutaneous wounds in 40 female Wistar rats were infected with *S. aureus* and then randomly divided into four groups: infected-wounded skin treated with sterile saline solution (control group), treated with ultrasound (ultrasound group), treated by a mixture of MINO and HA (MINO+HA group) and treated with a mixture of MINO and HA combined with ultrasound (MINO+HA+ultrasound group). General observations of the wound healing were photographed. After three treatments, bacterial counts were obtained to determine antibacterial efficacy and wound healing was assessed by histological analysis and evaluation of inflammatory cytokine levels (TNF-*α* and IL-1*β*) by immunohistochemistry. Compared with the control group, both the MINO+HA group and the MINO+HA+ultrasound group achieved a significant wound square reduction of 43.7% and 54.9 %, respectively (*P*<0.001). A small number of inflammatory cells, organization of collagen fibres and maturation of granulation tissue were observed in the histological evaluation of the MINO+HA+ultrasound group. The expression levels of TNF-*α* and IL-1*β* in the MINO+HA+ultrasound group were decreased compared to both the control group and the MINO+HA group (*P*<0.001). These findings revealed the possibility of using a mixture of MINO and HA combined with ultrasound to minimize inflammation and promote tissue regeneration during the treatment of wound infections.

## Introduction

Skin, as an initial barrier, protects the human body against external stimuli, including pathogens, poisons and external injuries [1]. Infected skin wounds show dynamic pathological processes, usually starting immediately after the invasion of microbial pathogens [2]. Due to the diversity of pathogenic microorganisms, the control and care of wound infections is often challenging [3]. Wound infection can trigger complex cellular responses, including the activation of stress signals and an imbalance of inflammatory processes [4].

*Staphylococcus aureus* is a Gram-positive bacterium and one of the most common pathogens in humans [5]. *S. aureus* proliferates at the site of infection, secretes a variety of toxins, enzymes, proteins and forms bacterial biofilms [6]. The extracellular polymeric substances of biofilms serve as a protective barrier for bacteria, shielding them from immune cells and various environmental stresses [7]. However, the presence of exotoxins within the biofilm triggers an immune response, ultimately resulting in pus formation and tissue necrosis [8]. Cytokines, like TNF-*α*, IL-1, IL-6 and C-reactive protein, are produced during wound infection and act as key indicators of inflammation after injury [9]. Tracking these crucial biomarkers can help to evaluate the intensity and progression of inflammation. Surgical debridement is a common treatment for wound infection. Surgical debridement may seem to completely clear the infection, but it may lead to damage to surrounding normal tissues, induce scar formation and secondary infection. The other traditional treatment of wound infection is the application of antibiotics. Minocycline (MINO) is a second-generation semi-synthetic tetracycline antibiotic with a broad spectrum of antimicrobial activity [10]. It is effective against *S. aureus*, Gram-negative bacteria and anaerobic bacteria by inhibiting bacterial protein synthesis [11]. In addition, MINO has anti-inflammatory effects that are completely independent of its antimicrobial properties [12]. It can alleviate inflammation by suppressing the release of inflammatory cytokines such as IL-6, IL-1 and TNF-*α* [13]. Although it can control infection, long-term application of antibiotics may cause gastrointestinal discomfort, liver and kidney function damage and bacterial resistance [14]. It is crucial to find a therapeutic strategy that can shorten the antibiotic treatment cycle and improve efficacy.

Hyaluronic acid (HA) is a natural polysaccharide mainly composed of *N*-acetylglucosamine and glucuronic acid. HA has excellent biocompatibility, low toxicity and biodegradability, which can promote cell migration and proliferation and help accelerate wound healing and repair [15]. Its anti-inflammatory effect is achieved by reducing the release of inflammatory cytokines, thereby reducing tissue damage and further promoting wound healing [16]. HA can enhance hydration and increase drug permeability through its high osmotic pressure properties [17].

Low-intensity ultrasound, as a novel non-invasive treatment method, has attracted increasing attention in recent years. Many experiments have shown that low-intensity ultrasound can enhance the bactericidal effect of antibiotics. Hou Y. *et al.* demonstrated that low-intensity ultrasound can enhance the permeability of biofilms to antibiotics [18]. When low-intensity ultrasound is combined with tobramycin, it can change the forms of biofilm. This study showed that low-intensity ultrasound can increase the permeability of cell walls, thereby enhancing the bactericidal effect of antibiotics on bacteria [19]. Our previous work demonstrated that HMME-mediated low-intensity ultrasound can efficiently prevent the proliferation of *S. aureus*. HMME-mediated low-intensity ultrasound is not only suitable for aerobic bacteria, but also can effectively inhibit the growth of anaerobic bacteria such as *Porphyromonas gingivalis*. In addition, HMME-mediated low-intensity ultrasound can also alleviate periodontal inflammation in rats and reduce alveolar bone loss.

The purpose of this study was to investigate whether the mixture of MINO and HA combined with ultrasound could reduce inflammation caused by *S. aureus* and promote tissue regeneration in the wound healing rat model.

## Methods

### Experimental animals

Forty female Wistar rats weighing between 200 g and 250 g were purchased from the animal experimental centre of the Second Affiliated Hospital of Harbin Medical University (Harbin, People’s Republic of China). They were kept in polyethylene plastic cages and were fed with free access to water and rat feed. All rats were acclimatized to the housing conditions for 1 week before the operative procedures. Animal care and experimental procedures were carried out in accordance with the National Institutes of Health guide for the care and use of Laboratory animals (NIH Publications Number 8023, revised 1978). The protocol was approved by the Experimental Animal Ethics Committee of Harbin Medical University.

### Bacterial strain and culture

*S. aureus* (ATCC 6538) were cultured in brain–heart infusion (BHI) (Difco, Detroit, MI, USA) broth in a constant temperature incubator with the temperature of 37 °C. After 48-h incubation, the bacterial suspensions were diluted with PBS and set to an optical density (A_630 nm_) of ~1×10^5^ cells ml^−1^.

### Induction of infected wounds

General anaesthesia was induced through an intraperitoneal injection of ketamine (80 mg kg^−1^) and dexmedetomidine (0.6 mg kg^−1^). A square cutaneous wound on the back of the rat was created by a surgical biopsy punch with a diameter of 12 mm. The wounds were inoculated with *S. aureus* at a concentration of 1×10^8^ cells ml^−1^ and administered every other day, totalling three times. The process above was operated by the same experienced operator (Deshu Zhuang). Then, the rats randomly received one of four treatments (10 rats per treatment): infected-wounded skin treated by sterile saline solution (control group), infected-wounded skin treated by ultrasound (ultrasound group), infected-wounded skin treated by a mixture of MINO and HA (MINO+HA group) and infected-wounded skin treated by a mixture of MINO and HA combined with ultrasound (MINO+HA+ultrasound group). All the treatments were applied every other day. The rats were euthanized after 5 days of treatment.

### MINO+HA preparation and ultrasound system

MINO at a concentration of 0.1% was purchased from a regular pharmacy to be used. HA hydrogel at a concentration of 1.5% was provided by Dr. Hui Biotechnology Co., Ltd. (Hangzhou, People’s Republic of China). MINO was dissolved in deionized water and then added to the HA hydrogel. The mixture was finally stirred at 37 °C completely and stored in a 4 °C refrigerator for later use.

The low-intensity ultrasound of 1 W cm^−2^ used in this experiment was provided by Harbin Institute of Technology (Harbin, People’s Republic of China). Ultrasonic intensities (W cm^−2^) were expressed as I^SPTP^ (spatial peak/temporal peak) measured 6 mm away from the ultrasonic transducer radiating surface in the degassed distilled water by a hydrophone (Onda Corp., Sunnyvale, CA, USA). In our experiment, the ultrasonic transducer (diameter, 6 mm; frequency, 1.0 MHz; duty factor, 10 %; pulse repetition frequency, 100 Hz) was placed over the wound regions with a connection of medical ultrasonic coupling agent for 300 s.

### General observation of the wound area

General observations including growth status, colour and texture of the wound healing. The wounds were photographed with a digital camera on days 0, 1, 3 and 5 after wound induction. The wound images were analysed by image software (ImageJ, National Institutes of Health, USA). The percentage of wound area was assessed as follows: percentage wound area = [wound area on day *X* / wound area on day 0] × 100.

### Bacterial extraction of the wound area

Bacterial samples of 5 rats per group were obtained from the epidermal surface immediately at the end of the treatments. For all the samples, dilution series (10^−1^–10^−8^) were made using sterile saline, and 100 µl of each dilution was applied to BHI agar at 37 °C. After cultivation for 2 days, the colonies growing on the agar were photographed, and the surviving bacteria numbers of the c.f.u. were determined automatically using the software ImageJ (version 1.51 r, National Institute of Health, USA).

### Haematoxylin and eosin and immunohistochemical staining

After the clinical observation, the infected-wound skin tissues from the 40 rats were collected and fixed in paraformaldehyde (10%) for 48 h. The samples were dehydrated with ethanol, made transparent with xylene and subsequently embedded in paraffin. Serial 4 µm paraffin sections were obtained and stained with haematoxylin and eosin (HE) and immunohistochemistry. The immunohistochemistry procedure was conducted as follows: after successively incubating with antigen retrieval solution (Shanghai Yaji Biotechnology Co. Ltd., Shanghai, China), the slides were incubated with the primary antibody (1L-1*β*/TNF-*α*) overnight at 4 °C. Then the slides were rinsed and incubated with the corresponding secondary antibody (Shanghai Yaji Biotechnology Co. Ltd.; Shanghai, China) for 30 min followed by 3,3′-diaminobenzidine staining. The sections were analysed and photographed using a Nikon Eclipse 80i microscope with QIcam camera (Burnaby, BC, Canada) and NIS Elements software (version 4.20, Nikon). All epidermal cells in a randomly selected field of view were counted and repeated until the total reached 1,000 cells. Then, the number of positive cells per 1,000 cells was calculated.

### Statistical data analysis

The data were statistically analysed using SPSS 22.0 software. Significant differences between the groups were determined with an ANOVA followed by Tukey’s post hoc test. The statistical significance was set at *P*<0.05. *P*-values under 0.001 were considered highly statistically significant.

## Results

### Visual observation and measurement of wound size

**Fig. 1.**
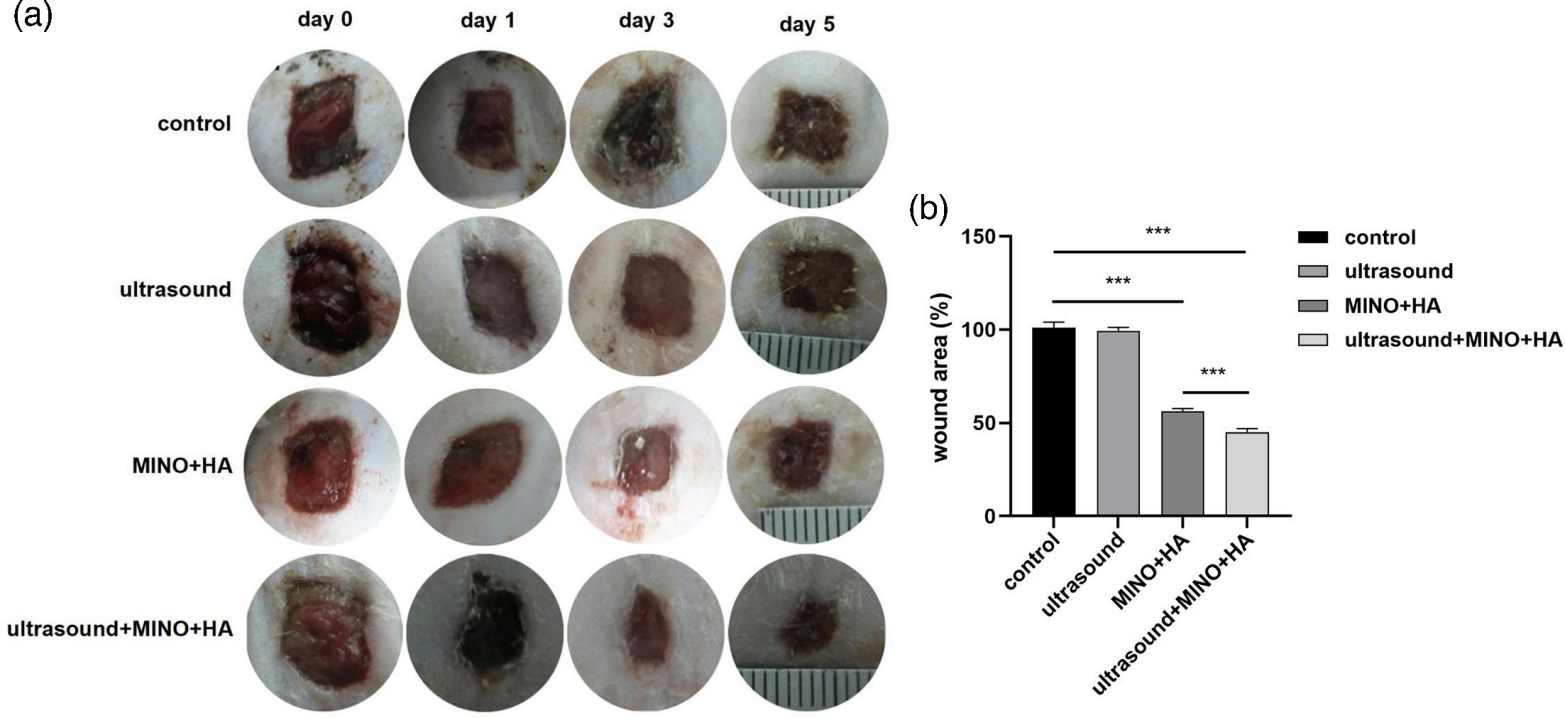
Wound area. (**a**) Photos of the wound area in each group at day 0, 1, 3 and 5. (**b**) Mean±sd of the data of the wound area in each group. Notes: *n*=10 in each group; *** indicates *P<*0.001.

Photographs of the infected wounds were taken on days 0, 1, 3 and 5 post-wound induction, as shown in Fig. 1a. On day 0, the wound surface exhibited an irregular contour, the perilesional skin appeared dark red and ulceration was observed at the wound margins. With the extension of treatment duration, it was observed that in the MINO+HA and MINO+HA+ultrasound groups, the wound area progressively diminished, the margins became well-defined and continuous and no obvious ulceration was found. In Fig. 1b, statistical analysis revealed that the wound area in MINO+HA and MINO+HA+ultrasound groups was significantly reduced compared with the control group (*P<*0.001). Compared to the MINO+HA group, the wound area in the MINO+HA+ultrasound group was significantly reduced (*P<*0.001). However, the wound area in the ultrasound group was 99.3, which did not exhibit statistically significant differences when compared to the control group (*P>*0.05).

### Antibacterial efficiency on wound infection

**Fig. 2.**
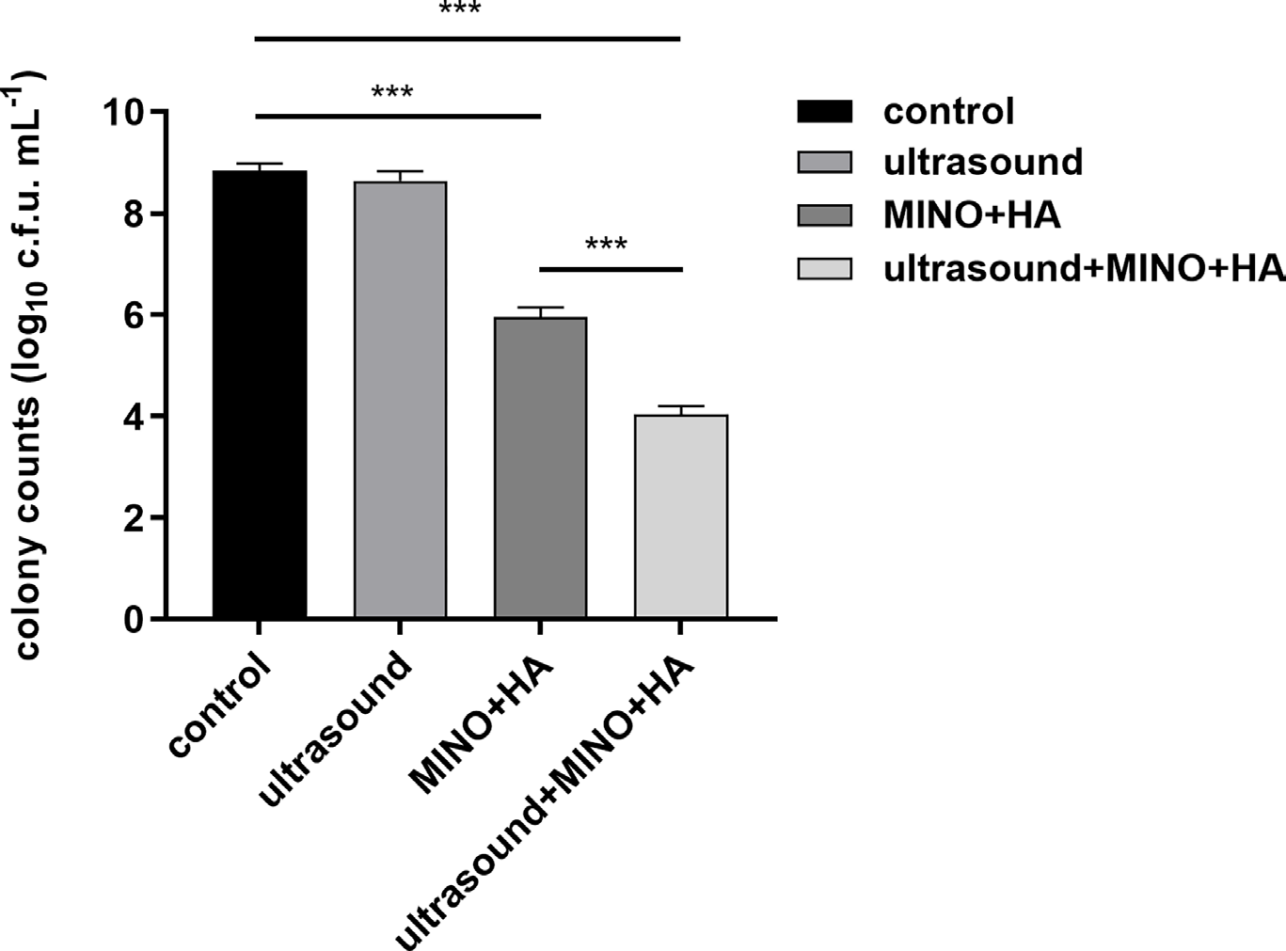
Bactericidal effect of MINO+HA+ultrasound on *S. aureus*-infected wounds in rats. Date represents mean values (*n*=10), and error bars represent sd. *** indicates *P*<0.001.

The antibacterial efficiency of MINO+HA+ultrasound on wound infection was demonstrated through c.f.u. per millilitre counts. As illustrated in Fig. 2, after treatment with MINO+HA and MINO+HA+ultrasound, bacterial growth was significantly decreased compared to the control group (*P*<0.001). Notably, a 1.9 log reduction in c.f.u. was observed when MINO+HA was combined with ultrasound in the treatment (*P*<0.001). However, there was no significant effect when treated with ultrasound alone (*P*>0.05).

### Histological analysis of the wound

**Fig. 3.**
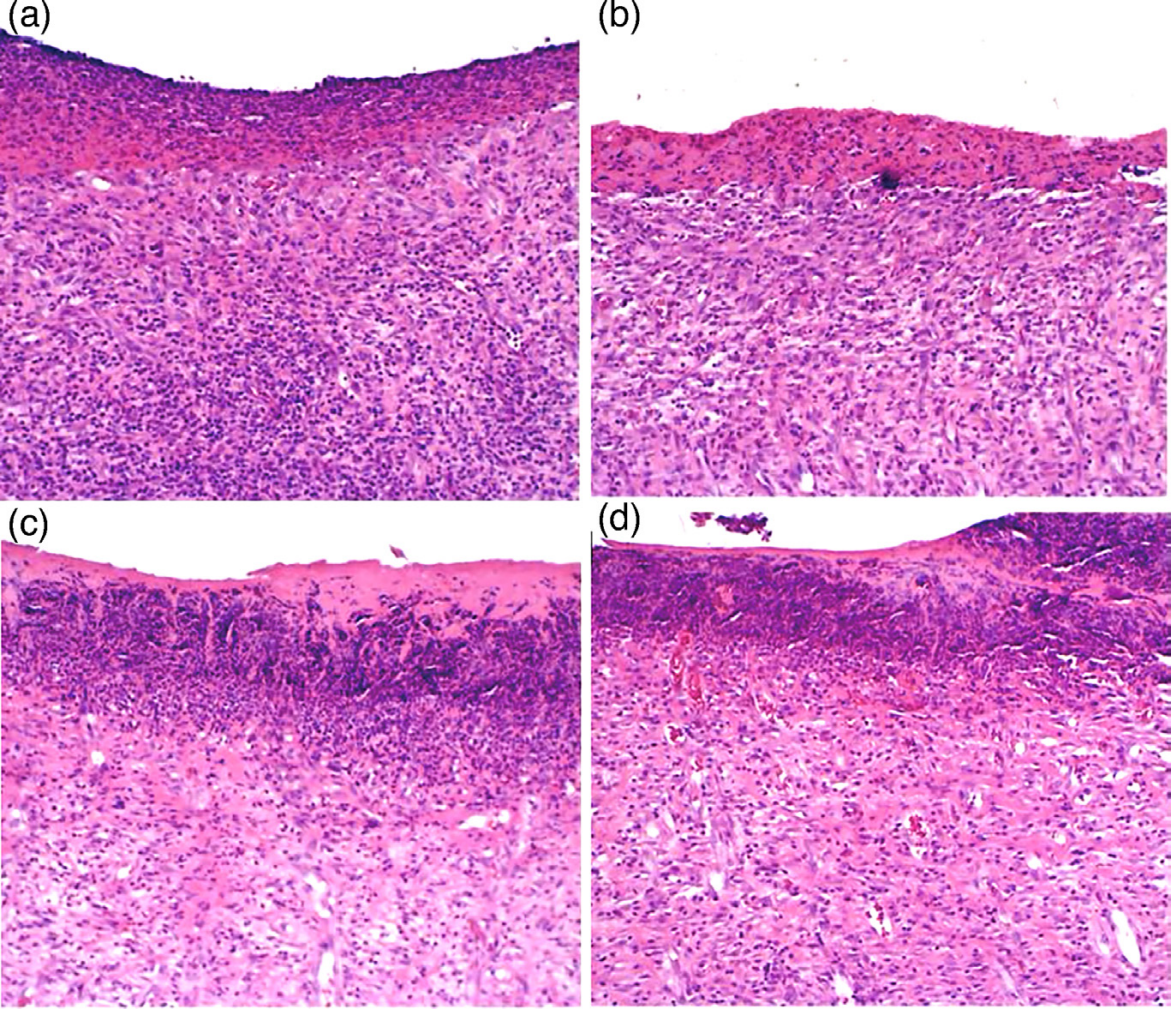
HE staining images of rat skin samples. (**a**) Control group (**b**) Ultrasound group (**c**) MINO+HA group (**d**) MINO+HA+ultrasound group. Original magnification for a, b, c and d was ×12.5.

The skin from the control and ultrasound groups showed an evident inflammatory infiltrate composed of polynuclear leukocytes. In addition, a thickening and disorganization of collagen fibres, a discontinuous epidermal layer and a minimal amount of granulation tissue as a result of the ulcer process were observed (Fig. 3a, b). While in the MINO+HA and MINO+HA+ultrasound groups, most specimens revealed organized collagen fibres with a moderate number of fibroblasts and a small number of macrophage and neutrophil infiltration. A substantial accumulation of blood cells was observed within the subcutaneous tissue. The granulation tissue demonstrated maturation without signs of ulceration (Fig. 3c, d).

### The expression of TNF-*α* in epidermis and dermis

**Fig. 4.**
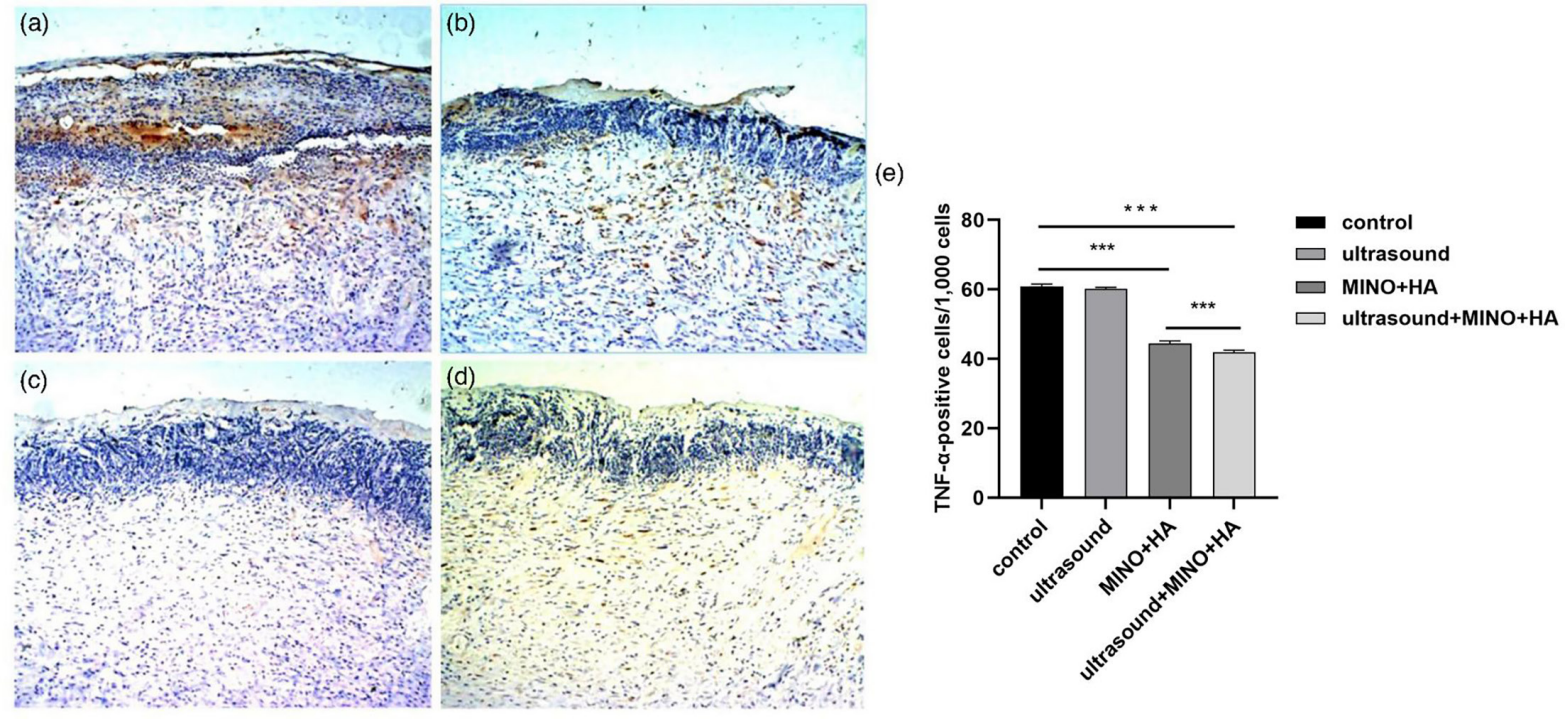
TNF-*α* immunohistochemical staining images of rat skin samples. (**a**) Control group, (**b**) ultrasound group, (**c**) MINO+HA group, (**d**) MINO+HA+ultrasound group. Original magnification for a, b, c and d was ×12.5. (**e**) Expression levels of TNF-*α* in rat skin in each group. Notes: *n*=10 in each group; *** indicates *P*<0.001.

Wound skin sections from the control group revealed extensive TNF-*α*-positive signals in the cytoplasm and intercellular substance of the epidermis and adjacent dermis, with strong TNF-*α* immunoreactivity noted on both sides of the basal cells (Fig. 4a). A moderate intensity of TNF-*α* positive signal was observed in the cytoplasm and intercellular substance of both the epidermis and dermis (Fig. 4b). In groups MINO+HA (Fig. 4c) and MINO+HA+ultrasound (Fig. 4d), TNF-*α* was predominantly expressed in the cytoplasm and intercellular substance of the dermis, with only a faint positive signal observed in the epidermis. The antigen preservation was evaluated by immunohistochemical staining, shown in Fig. 4e. The expression levels of TNF-*α* decreased in the MINO+HA and MINO+HA+ultrasound groups compared with the control group (*P*<0.001). There was no significant effect on the ultrasound group compared with the control group (*P*>0.05). It is important to highlight that the expression level of TNF-*α* in the MINO+HA+ultrasound group was decreased compared to both the control group and the MINO+HA group (*P*<0.001).

### The expression of IL-1*β* in epidermis and dermis

**Fig. 5.**
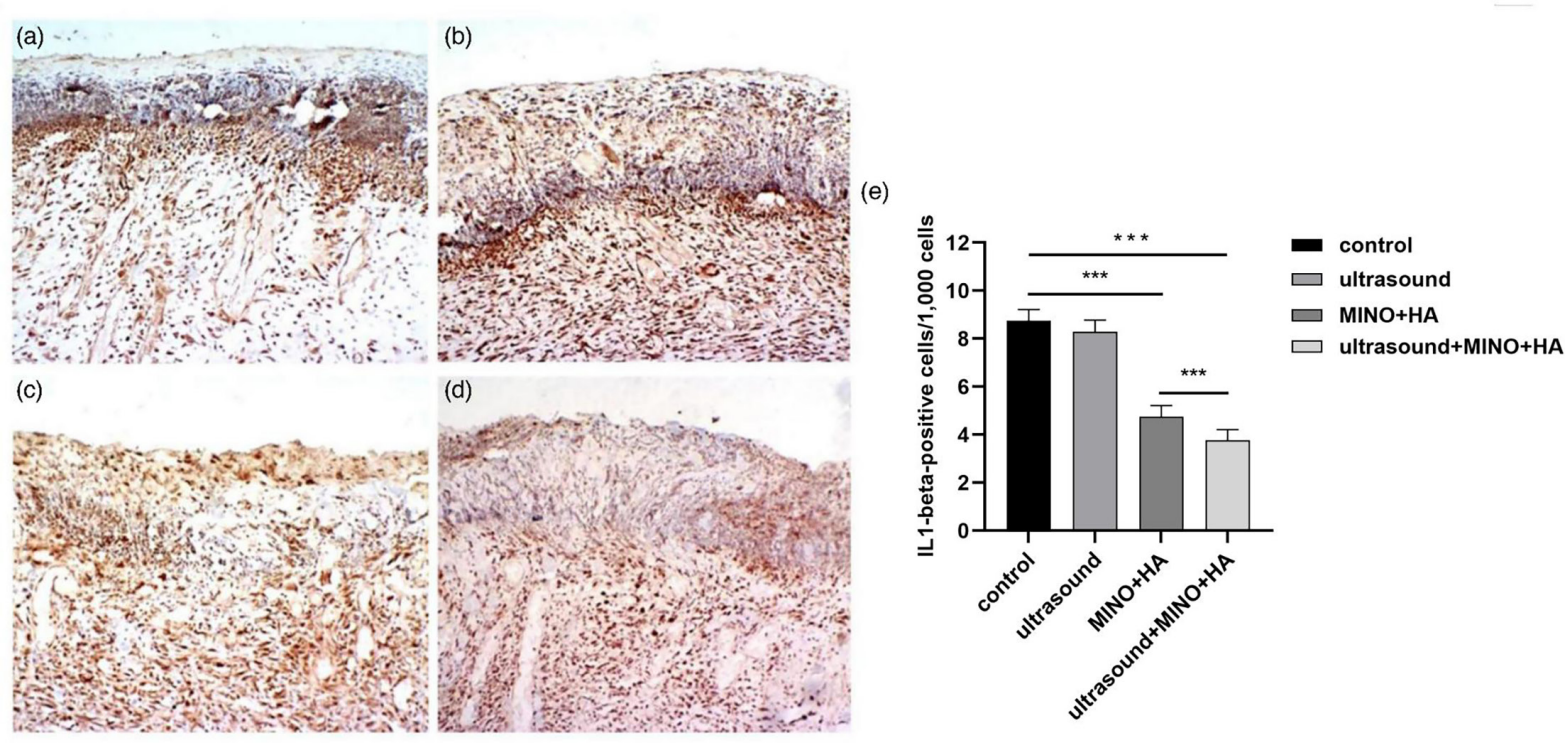
IL-1*β* immunohistochemical staining images of rat skin samples. (**a**) Control group, (**b**) ultrasound group, (**c**) MINO+HA group, (**d**) MINO+HA+ultrasound group. Original magnification for a, b, c and d was ×12.5. (**e**) Expression levels of IL-1*β* in rat skin in each group. Notes: *n*=10 in each group; *** indicates *P*<0.001.

In the control group, a minimal level of IL-1*β* expression was observed in the epidermis, whereas moderate IL-1*β* expression was detected in the dermis. In addition, the IL-1*β*-positive signals were obviously enhanced in the dermis adjacent to the basal layer (Fig. 5a). In both the epidermis and dermis, prominent and intense IL-1*β*-positive signals were detected in the ultrasound group (Fig. 5b). In groups MINO+HA (Fig. 5c) and MNO+HA+ultrasound (Fig. 5d), a faint IL-1*β*-positive signal was shown within the epidermal layer, while a moderate level of expression was observed in the dermis. Fig. 5e indicates that the MINO+HA and MINO+HA+ultrasound groups had a better capacity in reducing the antigen of IL-1*β* in comparison with using sterile saline solution (*P*<0.001). In addition, the expression level of IL-1*β* in the MINO+HA+ultrasound group was decreased compared to both the control group and the MINO+HA group (*P*<0.001).

## Discussion

Wound infection is a complex process involving the colonization and proliferation of bacterial pathogens on the wound surface, where they form biofilms. Biofilms protect bacteria from host immune cells, activate the host immune response and release inflammatory factors [3]. These factors cause blood vessels to dilate and increase vascular permeability, resulting in the infiltration of immune cells and plasma proteins into the tissue, which leads to redness and swelling [4]. Although inflammation initially serves a protective function, excessive inflammation can have detrimental effects. An accumulation of neutrophils, pathogens and fibrin forms pus, while proteinases activated by inflammatory factors degrade the extracellular matrix, disrupting tissue structure [5]. HA, a natural polysaccharide, has excellent biocompatibility and biodegradability, which promote cell migration and proliferation, enhance tissue hydration and accelerate wound healing [20]. When combined with antimicrobial peptides, it effectively disrupts bacterial signalling pathways, inhibits biofilm formation, suppresses the release of inflammatory factors and facilitates wound healing in infected tissues [21]. Furthermore, the combination of HA and azithromycin effectively destroys MRSA biofilms, demonstrating a potent therapeutic effect on bacterial infections [17]. Research has demonstrated that MINO exhibits strong antimicrobial effects against *S. aureus*, and no resistance to MINO was observed, thereby underscoring its potential for sustained long-term application [22]. Additionally, MINO is effective against methicillin-resistant *S. aureus* (MRSA) and can control MRSA growth by inhibiting bacterial protein synthesis [23]. In our study, the wound area in the MINO+HA and MINO+HA+ultrasound groups was significantly reduced compared with the control group (*P*<0.001). Compared to the MINO+HA group, the wound area in the MINO+HA+ultrasound group was significantly reduced (*P*<0.001). The wound edges became clearer and more continuous, and no obvious ulcers were observed. A 1.9 log reduction in c.f.u. was observed when MINO+HA was combined with ultrasound in the treatment (*P*<0.001). This suggests that the treatment not only effectively inhibited bacterial proliferation but also promoted tissue repair and regeneration.

Ultrasound therapy is non-invasive and radiation-free and enhances drug penetration [24]. Low-intensity ultrasound, when combined with antimicrobial peptides, facilitates their entry into bacterial cells to bind DNA, thereby exhibiting strong antibacterial activity against *S. aureus* [25]. Low-intensity ultrasound, mediated by hematoporphyrin monomethyl ether, can further penetrate MRSA biofilms [26]. In our study, low-intensity ultrasound enhanced the efficacy of MINO and HA by increasing drug permeability and improving blood circulation. Therefore, the combination of MINO, HA and low-intensity ultrasound achieves a synergistic effect, significantly enhancing wound healing. This synergism can be attributed to the following features of low-intensity ultrasound: singlet oxygen generation, cavitation, sonoluminescence and thermal effects. Singlet oxygen atoms, energized by ultrasound, transition to an excited state, potentially damaging mitochondria and DNA. Cavitation, which can be either inertial or non-inertial, generates reactive oxygen species and increases cell membrane permeability. Sonoluminescence refers to the emission of photon radiation from cavitation bubbles, which, along with the high temperature and pressure generated, induces oxidative stress within cells, amplifying cellular and bacterial damage. Thermal effects arise from the absorption of sound energy by tissues, converting it into heat, which promotes blood circulation, enhances drug diffusion and directly kills bacteria [24].

HA plays a crucial role in enhancing cell migration and collagen fibre formation, while MINO further supports tissue repair through its antibacterial and anti-inflammatory properties [27]. HA+MINO effectively inhibits the pro-inflammatory factor expression [22]. These findings are in line with our present study. After the treatment, bacterial growth was significantly decreased compared to the control group (*P*<0.001). Immunohistochemical results showed that the expression levels of TNF-*α* and IL-1*β* in the MINO+HA groups were significantly reduced (*P<0.001).* It is worth noting that a 1.9 log reduction in c.f.u. was observed when MINO+HA was combined with ultrasound in the treatment (*P<*0.001). However, there was no significant antibacterial effect when treated with ultrasound alone (*P*>0.05). From the immunohistochemistry results, the expression levels of TNF-*α* and IL-1*β* in the MINO+HA+ultrasound group were decreased compared to both the control group and the MINO+HA group (*P*<0.001). This may be due to the cavitation and penetration effects of low-intensity ultrasound [28]. Moreover, low-intensity ultrasound combined with betamethasone gel significantly increased anti-inflammatory activity. Studies have also demonstrated that HA and ultrasound together can slow the progression of osteoarthritis [29]. Low-intensity ultrasound may promote the penetration of MINO+HA by increasing tissue permeability, thus enhancing their antibacterial and anti-inflammatory effects.

## Conclusion

The combination of MINO, HA and low-intensity ultrasound significantly enhances wound healing and infection control. This multi-modal approach effectively inhibits bacterial proliferation, reduces inflammation and accelerates tissue repair, showing great potential for treating infected wounds.

## Abbreviations

BHI: brain–heart infusion
HA: hyaluronic acid
HE: haematoxylin and eosin
MINO: minocycline
MRSA: methicillin-resistant *Staphylococcus aureus*
